# Differential in vivo activation of monocyte subsets during low-grade inflammation through experimental endotoxemia in humans

**DOI:** 10.1038/srep30162

**Published:** 2016-07-22

**Authors:** B. Thaler, P. J. Hohensinner, K. A. Krychtiuk, P. Matzneller, L. Koller, M. Brekalo, G. Maurer, K. Huber, M. Zeitlinger, B. Jilma, J. Wojta, W. S. Speidl

**Affiliations:** 1 Department of Internal Medicine II, Medical University of Vienna, Vienna, Austria; 2 Ludwig Boltzmann Cluster for Cardiovascular Research, Vienna, Austria; 3 Department of Clinical Pharmacology, Medical University of Vienna, Vienna, Austria; 4 3rd Medical Department for Cardiology and Emergency Medicine, Wilhelminen Hospital, Vienna, Austria; 5 Core Facilities, Medical University of Vienna, Vienna, Austria

## Abstract

Human monocytes are a heterogeneous cell population, which can be divided into a classical (CD14++CD16−), a non-classical (CD14+CD16+), and an intermediate (CD14++CD16+) subset. We hypothesized that low-grade inflammation may differentially affect monocyte subsets. We used a human lipopolysaccharide (LPS) infusion model to mimic low-grade inflammation to identify, which monocyte subsets are preferentially activated under these conditions. Monocyte subsets were identified by staining for CD14 and CD16, activation status of monocytes was analyzed by staining for CD11b and a novel *in situ* mRNA hybridization approach to detect IL-6 and IL-8 specific mRNA at the single-cell level by flow cytometry. After LPS challenge, cell numbers of monocyte subsets dropped after 2 h with cell numbers recovering after 6 h. Distribution of monocyte subsets was skewed dramatically towards the intermediate subset after 24 h. Furthermore, intermediate monocytes displayed the largest increase of CD11b expression after 2 h. Finally, IL-6 and IL-8 mRNA levels increased in intermediate and non-classical monocytes after 6 h whereas these mRNA levels in classical monocytes changed only marginally. In conclusion, our data indicates that the main responding subset of monocytes to standardized low-grade inflammation induced by LPS in humans is the CD14++CD16+ intermediate subset followed by the CD14+CD16+ non-classical monocyte subset. Circulating classical monocytes showed comparably less reaction to LPS challenge *in vivo*.

Monocytes are bone marrow derived phagocytic cells primarily involved in the innate immune response, but also in other processes such as tissue repair and angiogenesis^1^. However, monocytes form a heterogeneous cell population with several identifiable subsets. Human monocyte subsets can be distinguished by the expression of CD14, a co-receptor for toll like receptor 4 (TLR4) and thus a crucial component of LPS signaling, and CD16, a low affinity Fc gamma receptor^2^. Human monocytes consist of three major subsets defined as CD14++CD16− for classical monocytes, CD14++CD16+ for intermediate monocytes and CD14+CD16+ for non-classical monocytes^3^. Each cell type has been associated with a certain function. Classical monocytes have a high phagocytic capacity while intermediate and non-classical monocytes represent further differentiated proinflammatory and proapoptotic cells^4^,^5^. Previously, human non-classical monocytes were suggested to have patrolling function but were reported not to be stimulated by cell surface toll like receptors^6^. This view was challenged by a report showing that *in vivo* activation of non-classical monocytes occurred in a humanized mouse model describing a similar activation pattern of CD16+ and CD16− monocytes^7^. In addition, Mukherjee *et al*. demonstrated that non classical monocytes react strongly to *in vitro* LPS challenge^8^. However, reactivity of subsets in humans *in vivo* remains to be explored.

An increasing number of mostly small observational studies have investigated if the distribution of monocyte subsets is related to clinical stages of disease progression in cardiovascular pathologies. Most of these studies have described higher levels of CD16+ monocytes to be associated with cardiovascular disease progression^9^. Apart from one study that reported that the CD16− monocytes predict cardiovascular events independent of other risk factors, recent evidence points towards an association of CD14++CD16+ intermediate monocytes with inflammatory cardiovascular conditions, sepsis or inflammatory disease^8^,^10^. We have recently shown that proatherogenic lipoprotein profiles were associated with increased levels of intermediate or non-classical monocytes^11^,^12^,^13^ and intermediate monocytes further predicted cardiovascular outcome in patients with chronic kidney disease^14^. In addition, in a study of more than 900 patients the CD14++CD16+ subset of monocytes independently predicted cardiovascular events^15^ and patients with stable and acute heart failure displayed increased levels of intermediate monocytes^16^. However, insight in molecular mechanisms that form the basis for these associations is limited as most of the experimental studies investigating such mechanisms were done either *in vitro* or in mice *in vivo*. Since *in vitro* results must only be translated into the *in vivo* setting with considerable caution, in particular as culture conditions in *in vitro* experiments may alter subset distribution and surface marker expression on these cells, and since a lack of consensus as to how the inflammatory capacity of murine and human monocyte subsets are comparable still exists, *in vivo* investigations studying possible differences in inflammatory activation patterns of human monocyte subsets are warranted^1^.

We hypothesized that exposure of monocytes to inflammatory conditions may differentially affect the activation pattern of the three monocyte subsets *in vivo*. Therefore we analyzed the activation pattern of human monocyte subsets by measuring their expression of two classical cytokines, namely the classical inflammatory cytokine interleukin-6 (IL-6) and the classical chemokine IL-8 in a well-standardized human endotoxemia model of low-grade systemic inflammation^17^,^18^,^19^. We have chosen to measure IL-6 and IL-8 in our study as both interleukins are produced by monocytes and are critically involved in the pathogenesis of conditions characterized by an inflammatory state^20^,^21^,^22^,^23^. In addition, levels of IL-6 and IL-8 have been used previously to distinguish monocyte subsets after LPS stimulation^5^,^6^. Our study provides evidence that the main responding monocytes in humans *in vivo* are CD16+ monocytes, whereas circulating classical monocytes (CD14++CD16−) did not show strong reactivity under these conditions.

## Results

**Figure 1:**
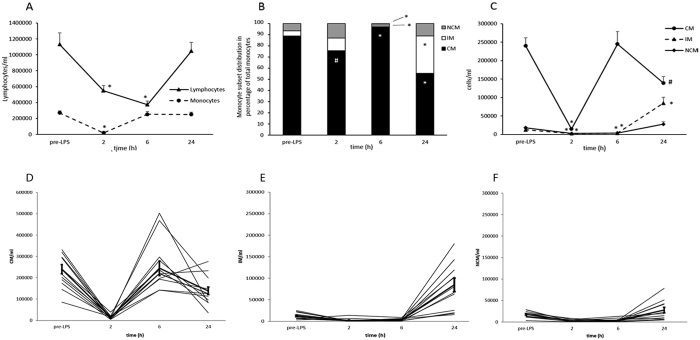
(**A**) Total lymphocyte and monocyte cell counts during human endotoxemia. Lymphocytes and monocytes were identified in a CD45/SSC scatter plot and followed over the given time course. Total cell number was calculated using 123 count eBeads as provided in Methods section. Total lymphocyte cell numbers dropped significantly 2 h and 6 h after LPS infusion with a return to baseline after 24 h. In contrast monocyte numbers dropped significantly after 2 h whereas total cell number was reverted to baseline already 6 h after LPS infusion. *p < 0.001 versus baseline. Values are given as average of cell number ± S.E.M. (**B**) Monocyte subset distribution during endotoxemia. Monocyte subsets are given according to their percentage distribution. After 2 h CM subset distribution was reduced compared to baseline, whereas IM and NCM showed a trend towards an increase. After 6 h, the monocytic pool consisted almost exclusively of CM, whereas after 24 h the distribution was shifted towards IM. *p < 0.001 versus baseline, ^#^p < 0.05 versus baseline. Values are given as average percent distribution. (**C**) Total monocyte subset cell counts during human endotoxemia. Monocyte subsets were identified as described in the Methods section. All three monocyte subsets displayed strong cell count reductions 2 h after endotoxemia induction. Cell numbers returned to baseline for CM after 6 h, whereas they remained reduced for IM and NCM. After 24 h total cell numbers were skewed towards IM as total cell numbers were increased for IM and decreased for CM. *p ≤ 0.001 versus baseline, ^#^p < 0.05 versus baseline. Values are given as average of cell number ± S.E.M. (**D**–**F**) Individual patient data of monocyte subset count for CM (**D**), IM (**E**) and NCM (**F**). Mean values ± S.E.M. are represented by bold lines.

Infusion of 2 ng/kg LPS induced monocytopenia and lymphocytopenia after 2 h and 6 h with a recovery of cell numbers after 24 h (Fig. 1A), which is consistent with previous reports^24^. Within our study we addressed the question of distribution of monocyte subsets during endotoxemia. 2 h after LPS injection, total cell numbers for all monocyte subsets dramatically dropped and were a fraction of the initial circulating monocyte numbers (Fig. 1B). For classical monocytes, we observed a drop to 5% of the initial count (p < 0.001), intermediate monocytes were reduced to 17% (p = 0.001) and non-classical monocytes were reduced to 13% (p < 0.001). 6 h after LPS injection, classical monocytes recovered to their initial cell number whereas intermediate and non-classical monocytes remained reduced. Interestingly, 24 h after endotoxemia induction, classical monocyte counts were reduced to 57% of the initial cell number before LPS injection (p < 0.05) and no significant change was observed for non-classical monocytes. In contrast, cell counts increased dramatically for intermediate monocytes (572% increase, p < 0.001). This massive increase is represented in the skewing of the monocyte subpopulation distribution towards the intermediate monocyte subset (Fig. 1C).

**Figure 2:**
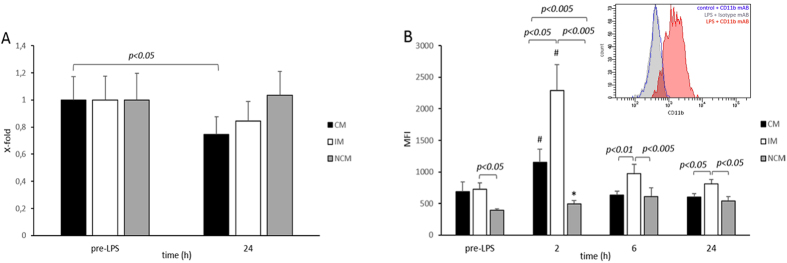
(**A**) Change in CD14 receptor surface density. CD14 cell surface density was evaluated in CM, IM, and NCM before LPS injection and 24 h after. CM monocytes showed a significant reduction in CD14 surface availability compared to base line, whereas IM and CM receptor density did not change significantly. Values are given as x-fold control ± S.E.M. (**B**) CD11b surface density on monocytes in human endotoxemia. Receptor density at baseline was similar in IM and CM and lowest in NCM. Two hours after LPS injection, CD11b values increased the most in IM followed by CM, and increased slightly in NCM. Comparing the individual subsets at 2 h revealed the highest CD11b surface availability for IM followed by CM and NCM. After 6 h CD11b values dropped significantly for CM and IM whereas they remained at a similar level in NCM. However, the IM subset remained the subset with the highest receptor density. A similar CD11b distribution was observed for 6 h and 24 h. *p < 0.001 versus baseline, ^#^p < 0.05 versus baseline. Values are given as mean fluorescence intensity ± S.E.M. The inset to (**B**) shows a representative histogram of classical monocytes treated for 4 h with or without LPS and stained with either an antibody against CD11b or the respective isotype control as outlined in the Methods section.

Classical monocytes were described to lose CD14 surface expression after exposure to LPS in a humanized mouse model^7^. Twenty-four hours after LPS infusion, classical monocytes showed a 22% (p < 0.05) reduction of mean fluorescence intensity of CD14 expression, whereas a slight, albeit not significant increase of CD14 expression was seen in non-classical monocytes in human test subjects. A slight but not significant decrease in CD14 was seen in intermediate monocytes (Fig. 2A).

To determine the effect of LPS activation on monocyte subsets we analyzed the surface expression of activated CD11b, a member of the β2 integrin family. Mild endotoxemia induces a rapid increase in CD11b surface expression on the total monocyte population^25^. The current study confirms these data and shows a similar CD11b up-regulation on the surface of the total monocyte population as quantified by mean fluorescence intensity (MFI) (2 h, 96% increase, p < 0.001). Whereas classical monocytes and non-classical monocytes only moderately increased CD11b surface expression to 176 ± 18% (p < 0.05) and 125 ± 7% (p < 0.001) of the baseline MFI value, respectively, intermediate monocytes displayed an increase to 328 ± 149% (p < 0.05) over baseline MFI. CD11b expression dropped to baseline levels after 6 h, albeit intermediate monocytes remained the cell subtype with the highest CD11b levels thereafter (Fig. 2B). As can be seen from the inset to Fig. 2B, staining for activated CD11b was specific as an isotype control antibody did not stain monocytes after 4 h of LPS treatment.

**Figure 3:**
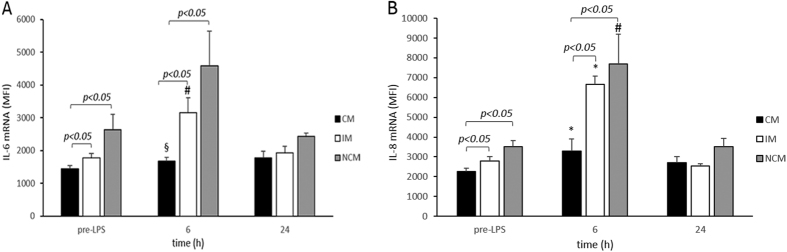
(**A**) IL-6 mRNA in monocytes subsets in human endotoxemia. IL-6 mRNA was determined using an *in situ* mRNA hybridization approach at the single-cell level by flow cytometry. Baseline mRNA levels were significantly higher in IM and NCM compared to CM. 6 h after LPS induction a marked increase of IL-6 mRNA in IM and NCM over CM was identified reverting to baseline conditions after 24 h. MFI denotes mean fluorescence intensity. ^§^p < 0.01 versus baseline, ^#^p < 0.05 versus baseline. Values are given as mean fluorescence intensity ± S.E.M. (**B**) IL-8 mRNA in monocytes subsets in human endotoxemia. IL-8 mRNA was determined using an *in situ* mRNA hybridization approach at the single-cell level by flow cytometry. Baseline mRNA levels were significantly higher in IM and NCM compared to CM. 6 h after LPS induction a marked increase of IL-8 mRNA in IM and NCM over CM was identified reverting to baseline conditions after 24 h. *p < 0.001 versus baseline, ^#^p < 0.05 versus baseline. Values are given as mean fluorescence intensity ± S.E.M.

Lastly, we compared cytokine activation patterns in monocyte subsets after LPS injection. To avoid misleading results with intracellular cytokine staining due to secretion of proteins we decided to use a novel *in situ* mRNA hybridization approach to detect IL-6 and IL-8 mRNA at the single-cell level by flow cytometry^26^. Overall IL-6 mRNA and IL-8 mRNA MFI were already slightly increased in intermediate and non-classical monocytes at baseline. LPS infusion increased IL-6 mRNA MFI levels to 180 ± 30% (p < 0.05) and 225 ± 80% (p = 0.12) and IL-8 mRNA MFI levels to 240 ± 14% (p < 0.001) and 232 ± 53% (p < 0.05) in intermediate and non-classical monocytes 6 h after LPS infusion respectively, whereas these levels increased only slightly to 119 ± 13% (p < 0.01) and 142 ± 18% (p < 0.001) in classical monocytes. After 24 h, MFI mRNA levels for IL-6 and IL-8 mRNA returned back to baseline in intermediate and non-classical monocytes suggesting no prolonged activation of circulating monocytes (Fig. 3A,B). Plasma levels for IL-6 and IL-8 in the same volunteers followed similar kinetics. They increased from non-detectable levels before LPS infusion to 1.8 ± 0.7 pg/ml IL-6 and to 31.3 ± 12.8 pg/ml IL-8 8 h after the infusion and returned to non-detectable levels at 24 h.

## Discussion

It is generally accepted that low-grade inflammation is associated with coronary artery disease caused by the presence of atherosclerotic lesions in the vessel wall^27^. Strong clinical and experimental evidence supports the notion that the innate immune system in general and monocytes and monocyte-derived macrophages in particular are key players in the development and progression of such lesions^28^.

Human monocytes are not a homogeneous cell population. Based on their expression of the surface markers CD14 and CD16 three major subsets defined as CD14++CD16− for classical monocytes, CD14++CD16+ for intermediate monocytes and CD14+CD16+ for non-classical monocytes can be distinguished in men^3^. Several clinical studies point towards an association of CD16+ subsets with pathologies characterized by a chronic inflammatory state such as coronary artery disease, obesity, arthritis, inflammatory diseases of the intestinal tract or systemic lupus erythematous^8^,^29^,^30^,^31^,^32^.

The exact role and response of monocyte subsets under inflammatory conditions and the underlying molecular mechanisms are currently critically discussed with diverging results from various *in vitro* studies. Most *in vivo* studies, on the other hand, addressing this subject, used murine models. Murine monocyte subsets, however, differ substantially from human monocyte subsets in their physiological and pathophysiological properties^1^,^3^. In our study we have used a well-characterized model of human LPS-induced low-grade inflammation to study the response of different monocyte subsets to inflammatory activation *in vivo* in men^33^,^34^.

In line with previous results, demonstrating an expansion of CD16+ monocytes in septic conditions *in vivo* and *in vitro*^8^,^35^,^36^, we find, after an initial drop in total monocyte numbers shortly after LPS-infusion and a subsequent recovery in total monocyte numbers to pre-LPS levels, an increase in intermediate and a decrease in classical monocytes 24 hours after LPS infusion. We extend the findings of Fingerle *et al*. and Dominguez-Nieto *et al*.^35^,^36^ showing that this effect occurs early and even during low-grade inflammation. Still, cautious interpretation of data obtained from *in vivo* LPS studies is required as activated monocytes might disappear from peripheral blood as they adhere to the endothelium and therefore might subsequently be lost for sampling. However, the relative increase in intermediate monocytes in our study occurs after 24 hours and classical monocytes reappear already after 6 hours when the other two subsets are still clearly missing in the circulation. Furthermore it would be of interest if intermediate monocytes after LPS challenge originate directly from bone marrow or are derived from classical monocytes present in the circulation in this setting. In that respect it is noteworthy that Mandl *et al*. have shown recently that the human bone marrow contains a monocyte pool resembling intermediate monocytes^37^. In contrast Rogacev *et al*. found that the first monocytes that appear in peripheral blood of patients after myoablation and subsequent bone marrow transplantation are classical monocytes, followed by intermediate and then non-classical monocytes^38^. Although we cannot clarify the exact origin of intermediate monocytes in this study, we speculate that the intermediate subset might be a transitional subset bridging between the classical and non-classical population^5^ because endotoxemia reduces the surface density of the CD14 receptor together with an observed increase in the intermediate subset population in our study. However, we cannot rule out a mobilization of a CD16+ monocytic subfraction from the bone marrow.

Further changes in monocyte subsets were observed in regards to the adhesion protein CD11b. CD11b dimerizes with CD18 and is necessary for proper monocyte locomotion on the endothelial layer^39^. In addition CD11b can also act as a receptor for LPS necessary for LPS uptake^40^. Previously, mild endotoxemia in human subjects was shown to induce CD11b surface expression^25^. Analyzing subset specific activated CD11b surface availability we found that activated CD11b is predominantly induced in intermediate monocytes. CD11b expression patterns after LPS infusion suggest that classical monocytes are only weakly responsive to LPS *in vivo*. The increase in activated CD11b expression in intermediate cells could reflect a different adhesion pattern of intermediate cells upon inflammatory activation *in vivo*. Our data might support the suggested patrolling behavior of non-classical monocytes even after LPS stimulation as activated CD11b fluorescence intensity was lowest for non-classical monocytes. Of note, increased adhesion molecule receptor expression was reported for intermediate monocytes in ischaemic heart disease^41^ indicating the increased reactivity of intermediate cells towards an inflammatory stimulus.

The current paradigm for human monocytes is that classical monocytes react the most to LPS-induced inflammatory activation with a decrease in reactivity for intermediate and non-classical monocytes^5^. In addition, non-classical monocytes are viewed as the subset patrolling the blood stream with only a reduced reaction to inflammatory stimuli^6^. Our results, however, indicate that cytokine production in response to an inflammatory stimulus is enhanced in CD16+ monocyte subsets. *In vivo* exposure to LPS increased IL-6 and IL-8 mRNA expression in both intermediate and non-classical monocytes strongly, whereas only a minor effect was detectable for classical monocytes. This is especially striking when comparing RNA levels 6 h after LPS infusion between subsets, as both intermediate and non-classical monocytes show increased levels of at least 1.8 fold for IL-6 and more than 2 fold for IL-8. It should be noted that we have chosen to perform *in situ* hybridisation as a more reliable approach to quantitate IL-6 and IL-8 production in these cells as these two cytokines are usually secreted and thus intracellular protein levels most likely do not reflect the actual amount of proteins produced by the cell. Plasma levels for IL-6 and IL-8 in the same volunteers followed similar kinetics reaching peak levels 8 h after LPS infusion. However, the rather low numbers of intermediate and non-classical monocytes early after LPS infusion might limit their contribution to overall plasma levels of IL-6 and IL-8 at that time period. Still it is tempting to speculate that these two subsets of monocytes might contribute to local IL-6 and IL-8 levels. In patients with a disease connected with an increase of CD16+ monocytes, the increased responsiveness of these subsets to inflammation might however be of more importance compared to healthy human subjects. Further work needs to clarify whether this increased reaction of intermediate and non-classical monocytes is due to a different signaling transduction due to CD16 availability as already suggested by Shalova *et al*. who have shown that CD16 regulates the TRIF-dependent TLR4 signalling in monocyte^42^. Interestingly, previous reports also suggested an increase of TLR4 receptor in CD16+ cells, which might contribute to the observed behavior^43^.

In conclusion, we demonstrate in this report for the first time in human subjects that *in vivo* inflammatory activation of monocytes is occurring mainly in the CD16+ monocyte fraction of intermediate and non-classical monocytes. Intermediate monocytes are the key responders to low-grade inflammation represented by an increased expression of the integrin CD11b and the inflammatory cytokines IL-6 and IL-8. Non-classical monocytes upregulated IL-6 and IL-8 but did not show massive changes in CD11b surface expression. This *in vivo* behavior is in contrast to previously reported *in vitro* behavior of isolated non-classical monocytes^6^. On the contrary, classical monocytes displayed only slight changes in mRNA levels of IL-6 or IL-8, and responded with a downregulation of CD14 surface availability. Our results might indicate a prominent *in vivo* role for CD16 in TLR4 signaling. Whether this role might be due to changes in downstream TLR4 signaling pathways as suggested previously^42^ needs to be further addressed. Overall, our data suggest a predominant role for intermediate and non-classical monocyte subsets in the course of human low-grade inflammation. This observation is in so far interesting, as an increase of CD16+ intermediate monocytes has been described as characteristic for cardiovascular disease^16^. In addition in a recent study of more than 900 patients the CD14++CD16+ subset of monocytes independently predicted cardiovascular events^15^. Our results lead us to speculate that the intermediate subset of monocytes could represent a possible target for the development of new therapeutic strategies in the treatment of conditions characterized by an inflammatory state and the involvement of the innate immune system.

## Methods

### Endotoxin model

This study was approved by the Ethical Committee of the Medical University of Vienna and complies with the Declaration of Helsinki. Informed consent was obtained from all participants. Healthy volunteers (n = 12) were injected with a bolus infusion of LPS (2 ng/kg; CCRE lot from NIH)^33^. Blood samples were obtained before LPS injection and at 2 h, 6 h, 8 h and 24 h after injection. Study participants were apparently healthy with no symptoms of relevant clinical illness or medication within the last 3 weeks before inclusion. In addition, no blood donation was allowed within the last 4 weeks before inclusion. Furthermore, volunteers had no liver or kidney dysfunction and passed a physical examination with normal status for general appearance, head, ears, throat, nose, lymph nodes, cardiovascular, respiratory, abdomen, musculoskeletal, and neurologic status. Mean age of participants was 28.7 years. One proband was smoking <5 cigarettes per day. The remaining eleven were non-smokers.

### FACS analysis

Whole blood samples were stained for CD45 (PerCP, Clone SD1, eBiosciences, CA, USA), CD14 (PeCy7, Clone 61D3, eBiosciences), CD16 (APC-H7, Clone 3G8, BD Biosciences, CA, USA) and CD11b (FITC, Clone CBRM1/5, eBiosciences) and incubated for 15 min at room temperature. Antibodies were used in the concentrations recommended by the respective company. In addition, specificity of the CD11b antibody was tested with an isotype control (eBiosciences), which was used to validate the binding to the activation-specific epitope of CD11b.

**Figure 4:**
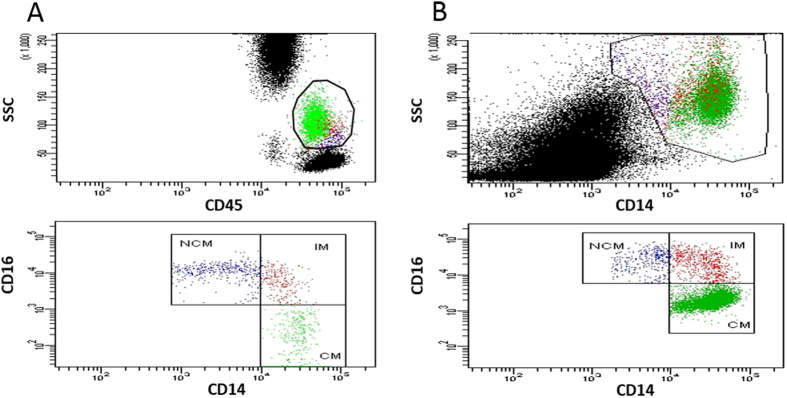
(**A**) Gating strategy for identification of classical (CM), intermediate (IM), and non-classical (NCM) monocytes. After whole blood staining and red blood cell lysis, the monocyte population was defined as CD45 positive cells exhibiting a typical location in a CD45 and sideward scatter (SSC) scatter plot. The remaining CD45 population was then distinguished according to their CD14 and CD16 surface expression identifying them as CM, IM and NCM. (**B**) Gating strategy used for *in situ* hybridization experiments to identify classical (CM), intermediate (IM), and non-classical (NCM) monocytes. After isolation of peripheral blood mononuclear cells via density gradient centrifugation, the monocyte population was defined as CD14 positive cells and monocyte subsets were then defined according to their CD14 and CD16 expression.

1 ml of FACS lysing solution (BD Biosciences) was added to lyse red blood cells and samples were incubated for another 15 min. Afterwards, cells were washed three times with phosphate buffered saline (PBS), ph = 7.4 + 1% bovine serum albumin (BSA; Sigma, USA) and acquired. 100.000 events were recorded per sample and analyzed on a BD FACS Canto II using FACS DIVA software. Gating strategy can be seen in Fig. 4A. Absolute cell numbers were determined using 123 count ebeads (Affymetrix, CA, USA) by adding 100 μl of the bead suspension directly to the sample before acquisition. 123 count ebeads were identified in a scatter blot using the fluorescent parameters FITC and PE and were used as a reference to calculate absolute cell numbers. Compensation was achieved by using single stains of BD CompBead beads (BD Biosciences) with the respective antibodies.

### *In situ* hybridization for IL-6 and IL-8

Peripheral blood mononuclear cells were isolated from whole blood via Ficoll centrifugation (GE Healthcare, IL, USA) and stained for CD14 and CD16. Afterwards, samples were washed once with PBS + 1% BSA, fixed for 30 min at 4 °C with PrimeFlow RNA Fixation Buffer (Affymetrix) and permeabilized with Permeabilization Buffer (Affymetrix) for another 30 min at 4 °C. *In situ* hybridization for IL-6 and IL-8 was performed using Prime FlowRNA assay kit and probe sets according to the manufacturer (Affymetrix)^26^. In short, samples were incubated with the appropriate target probes for 2 h at 40 °C, washed twice with PrimeFlow RNA Wash Buffer, resuspended in Storage Buffer and acquired on a FACS Canto II (BD Biosciences). Gating strategy is displayed in Fig. 4B. In short, 100.000 events per sample were recorded and the monocyte population was gated using CD14 as a selection marker. Afterwards monocyte subsets were evaluated according to the respective expression of CD16. Compensation was achieved by using single stains of BD CompBead beads with the respective secondary labels.

### Determination of IL-6 and IL-8 in plasma

IL-6 and IL-8 levels were determined in plasma samples of volunteers before, 8 h and 24 h after infusion of LPS using specific ELISAs (R&D Systems, MN, USA). The detection limit was 0.7 pg/ml for the IL-6 ELISA and 1.5 pg/ml for the IL-8 ELISA.

### Statistics

Sample size calculation revealed that we would need at least 10 patients to detect a difference of one standard deviation in pairwise comparison with a power of 80% and significance level (two-tailed) of 0.05^44^. Statistical analysis was performed using analysis of variance (ANOVA) with post-hoc testing according to Bonferroni and pairwise t-tests adjusted for multiple comparison using the method according to Bonferroni-Holm. Data is shown as mean ± standard error of the mean (SEM).

## Additional Information

**How to cite this article**: Thaler, B. *et al*. Differential in vivo activation of monocyte subsets during low-grade inflammation through experimental endotoxemia in humans. *Sci. Rep.* **6**, 30162; doi: 10.1038/srep30162 (2016).

## References

[b1] Ziegler-Heitbrock L. Monocyte subsets in man and other species. Cell. Immunol. 289, 135–139, 10.1016/j.cellimm.2014.03.019 (2014). 24791698

[b2] Hristov M. & Weber C. Differential role of monocyte subsets in atherosclerosis. Thromb. Haemost. 106, 757–762, 10.1160/TH11-07-0500 (2011). 21901241

[b3] Ziegler-Heitbrock L. . Nomenclature of monocytes and dendritic cells in blood. Blood 116, 80, 10.1182/blood-2010-02-258558 (2010). 20628149

[b4] Krychtiuk K. A., Kastl S. P., Speidl W. S. & Wojta J. Inflammation and coagulation in atherosclerosis. Hämostaseologie 33, 269–282, 10.5482/HAMO-13-07-0039 (2013). 24043155

[b5] Wong K. L. . Gene expression profiling reveals the defining features of the classical, intermediate, and nonclassical human monocyte subsets. Blood 118, 31, 10.1182/blood-2010-12-326355 (2011). 21653326

[b6] Cros J. . Human CD14dim monocytes patrol and sense nucleic acids and viruses via TLR7 and TLR8 receptors. Immunity 33, 375–386, 10.1016/j.immuni.2010.08.012 (2010). 20832340 PMC3063338

[b7] Aguilar-Ruiz S. R. . Human CD16+ and CD16− monocyte subsets display unique effector properties in inflammatory conditions *in vivo*. J. Leukoc. Biol. 90, 1119–1131, 10.1189/jlb.0111022 (2011). 21937707

[b8] Mukherjee R. . Non-Classical monocytes display inflammatory features: Validation in Sepsis and Systemic Lupus Erythematous. Sci. Rep. 5, 13886, 10.1038/srep13886 (2015). 26358827 PMC4566081

[b9] Libby P., Nahrendorf M. & Swirski F. K. Monocyte heterogeneity in cardiovascular disease. Semin. Immunopathol. 35, 553–562, 10.1007/s00281-013-0387-3 (2013). 23839097 PMC3755757

[b10] Berg K. E. . Elevated CD14++CD16− monocytes predict cardiovascular events. Circ. Cardiovasc. Genet. 5, 122–131, 10.1161/CIRCGENETICS.111.960385 (2012). 22238190

[b11] Krychtiuk K. A. . Association of Small Dense LDL Serum Levels and Circulating Monocyte Subsets in Stable Coronary Artery Disease. PloS one 10, 10.1371/journal.pone.0123367 (2015). PMC4388574 25849089

[b12] Krychtiuk K. A. . Small high-density lipoprotein is associated with monocyte subsets in stable coronary artery disease. Atherosclerosis 237, 589–596, 10.1016/j.atherosclerosis.2014.10.015 (2014). 25463093 PMC4270455

[b13] Krychtiuk K. A. . Monocyte Subset Distribution in Patients With Stable Atherosclerosis and Elevated Levels of Lipoprotein(a). J. Clin. Lipidol. 9, 533–541, 10.1016/j.jacl.2015.04.005 (2015). 26228671 PMC4533224

[b14] Rogacev K. S. . CD14++CD16+ monocytes and cardiovascular outcome in patients with chronic kidney disease. Eur. Heart J. 32, 84–92, 10.1093/eurheartj/ehq371 (2011). 20943670

[b15] Rogacev K. S. . CD14++CD16+ monocytes independently predict cardiovascular events: a cohort study of 951 patients referred for elective coronary angiography. J. Am. Coll. Cardiol. 60, 1512–1520, 10.1016/j.jacc.2012.07.019 (2012). 22999728

[b16] Wrigley B. J., Shantsila E., Tapp L. D. & Lip G. Y. CD14++CD16+ monocytes in patients with acute ischaemic heart failure. Eur. J. Clin. Invest. 43, 121–130, 10.1111/eci.12023 (2013). 23240665

[b17] Suffredini A. F. & Noveck R. J. Human endotoxin administration as an experimental model in drug development. Clin. Pharmacol. Ther. 96, 418–422, 10.1038/clpt.2014.146 (2014). 25236665

[b18] Mayr F. B. & Jilma B. Coagulation interventions in experimental human endotoxemia. Trans. Res. 148, 263–271, 10.1016/j.trsl.2006.08.002 (2006). 17145572

[b19] Mayr F. B. . Effects of low dose endotoxemia on endothelial progenitor cells in humans. Atherosclerosis 195, 6, 10.1016/j.atherosclerosis.2007.04.003 (2007). 17490672

[b20] Ataie-Kachoie P., Pourgholami M. H., Richardson D. R. & Morris D. L. Gene of the month: Interleukin 6 (IL-6). J. Clin. Pathol. 67, 932–937, 10.1136/jclinpath-2014-202493 (2014). 25031389

[b21] Rossi J.-F. F., Lu Z.-Y. Y., Jourdan M. & Klein B. Interleukin-6 as a therapeutic target. Clin. Cancer Res. 21, 1248–1257, 10.1158/1078-0432.CCR-14-2291 (2015). 25589616

[b22] Gales D., Clark C., Manne U. & Samuel T. The Chemokine CXCL8 in Carcinogenesis and Drug Response. ISRN Oncol. 2013, 859154, 10.1155/2013/859154 (2013). 24224100 PMC3810054

[b23] Campbell L. M., Maxwell P. J. & Waugh D. J. J. Rationale and Means to Target Pro-Inflammatory Interleukin-8 (CXCL8) Signaling in Cancer. Pharmaceuticals 6, 929–959, 10.3390/ph6080929 (2013). 24276377 PMC3817732

[b24] Jilma B. . Regulation of adhesion molecules during human endotoxemia. No acute effects of aspirin. Am. J. Respir. Crit. Care Med. 159, 857–863, 10.1164/ajrccm.159.3.9805087 (1999). 10051263

[b25] Lichte P. . Low dose LPS does not increase TLR4 expression on monocytes in a human *in vivo* model. Cytokine 63, 74–80, 10.1016/j.cyto.2013.04.014 (2013). 23673286

[b26] Porichis F. . High-throughput detection of miRNAs and gene-specific mRNA at the single-cell level by flow cytometry. Nat. Commun., 10.1038/ncomms6641 (2014). PMC4256720 25472703

[b27] Pearson T. A. . Markers of inflammation and cardiovascular disease: application to clinical and public health practice: A statement for healthcare professionals from the Centers for Disease Control and Prevention and the American Heart Association. Circulation 107, 499–511 (2003). 12551878 10.1161/01.cir.0000052939.59093.45

[b28] Oude Nijhuis M. M., van Keulen J. K., Pasterkamp G., Quax P. H. & de Kleijn D. P. Activation of the innate immune system in atherosclerotic disease. Curr. Pharm. Des. 13, 983–994 (2007). 17430162 10.2174/138161207780487593

[b29] Hristov M. & Heine G. H. Monocyte subsets in atherosclerosis. Hamostaseologie 35, 10.5482/HAMO-14-08-0030 (2014). 25396218

[b30] Poitou C. . CD14dimCD16+ and CD14+CD16+ monocytes in obesity and during weight loss: relationships with fat mass and subclinical atherosclerosis. Arterioscler. Thromb. Vasc. Biol. 31, 2322–2330, 10.1161/ATVBAHA.111.230979 (2011). 21799175

[b31] Weldon A. J. . Surface APRIL Is Elevated on Myeloid Cells and Is Associated with Disease Activity in Patients with Rheumatoid Arthritis. J. Rheumatol. 42, 749–759, 10.3899/jrheum.140630 (2015). 25729037 PMC4506783

[b32] Grip O., Bredberg A., Lindgren S. & Henriksson G. Increased subpopulations of CD16(+) and CD56(+) blood monocytes in patients with active Crohn’s disease. Inflamm. Bowel Dis. 13, 566–572, 10.1002/ibd.20025 (2007). 17260384

[b33] Jilma B. . Pharmacodynamics of active site-inhibited factor VIIa in endotoxin-induced coagulation in humans. Clin. Pharmacol. Ther. 72, 403–410, 10.1067/mcp.2002.127740 (2002). 12386642

[b34] Patel P. N., Shah R. Y., Ferguson J. F. & Reilly M. P. Human Experimental Endotoxemia in Modeling the Pathophysiology, Genomics, and Therapeutics of Innate Immunity in Complex Cardiometabolic Diseases. Arterioscler. Thromb. Vasc. Biol. 35, 525–534, 10.1161/ATVBAHA.114.304455 (2015). 25550206 PMC4344396

[b35] Fingerle G. . The novel subset of CD14+/CD16+ blood monocytes is expanded in sepsis patients. Blood 82, 3170–3176 (1993). 7693040

[b36] Domínguez-Nieto A. . Human endotoxin tolerance is associated with enrichment of the CD14+ CD16+ monocyte subset. Immunobiology 220, 147–153, 10.1016/j.imbio.2014.08.008 (2015). 25172544

[b37] Mandl M., Schmitz S., Weber C. & Hristov M. Characterization of the CD14++CD16+ monocyte population in human bone marrow. PloS one 9, 10.1371/journal.pone.0112140 (2014). PMC4219836 25369328

[b38] Rogacev K. S. . Immunosuppression and monocyte subsets. Nephrol. Dial. Transplant. 30, 143–153, 10.1093/ndt/gfu315 (2015). 25313167

[b39] Schenkel A. R., Mamdouh Z. & Muller W. A. Locomotion of monocytes on endothelium is a critical step during extravasation. Nat. Immunol. 5, 393–400, 10.1038/ni1051 (2004). 15021878

[b40] Scott M. J. & Billiar T. R. Beta2-integrin-induced p38 MAPK activation is a key mediator in the CD14/TLR4/MD2-dependent uptake of lipopolysaccharide by hepatocytes. J. Biol. Chem. 283, 29433–29446, 10.1074/jbc.M803905200 (2008). 18701460 PMC2570897

[b41] Wrigley B. J., Shantsila E., Tapp L. D. & Lip G. Y. Increased expression of cell adhesion molecule receptors on monocyte subsets in ischaemic heart failure. Thromb. Haemost. 110, 92–100, 10.1160/TH13-02-0088 (2013). 23740177

[b42] Shalova I. N. . CD16 regulates TRIF-dependent TLR4 response in human monocytes and their subsets. J. Immunol. 188, 3584–3593, 10.4049/jimmunol.1100244 (2012). 22427642

[b43] Skinner N. A., MacIsaac C. M., Hamilton J. A. & Visvanathan K. Regulation of Toll-like receptor (TLR)2 and TLR4 on CD14dimCD16+ monocytes in response to sepsis-related antigens. Clin. Exp. Immunol. 141, 270–278, 10.1111/j.1365-2249.2005.02839.x (2005). 15996191 PMC1809439

[b44] Dupont W. D. & Plummer W. D. Jr. Power and sample size calculations. A review and computer program. Control Clin. Trials 11, 116–128, 0197-2456(90)90005-M (1990). 2161310 10.1016/0197-2456(90)90005-m

